# Supplementary figures and images for: Protein phosphatase 1 in association with Bud14 inhibits mitotic exit in Saccharomyces cerevisiae
Source: eLife. 2021 Oct 11;10:e72833. doi: 10.7554/eLife.72833 (PMC8577847; doi:10.7554/eLife.72833)

Figure 3 - Figure Supplement 1A-Source Data 1

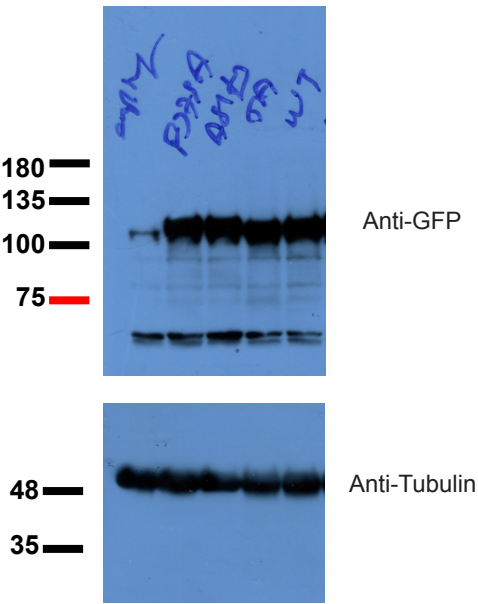

Supplement: Figure 3—figure supplement 1—source data 1. [file elife-72833-fig3-figsupp1-data1.pdf]

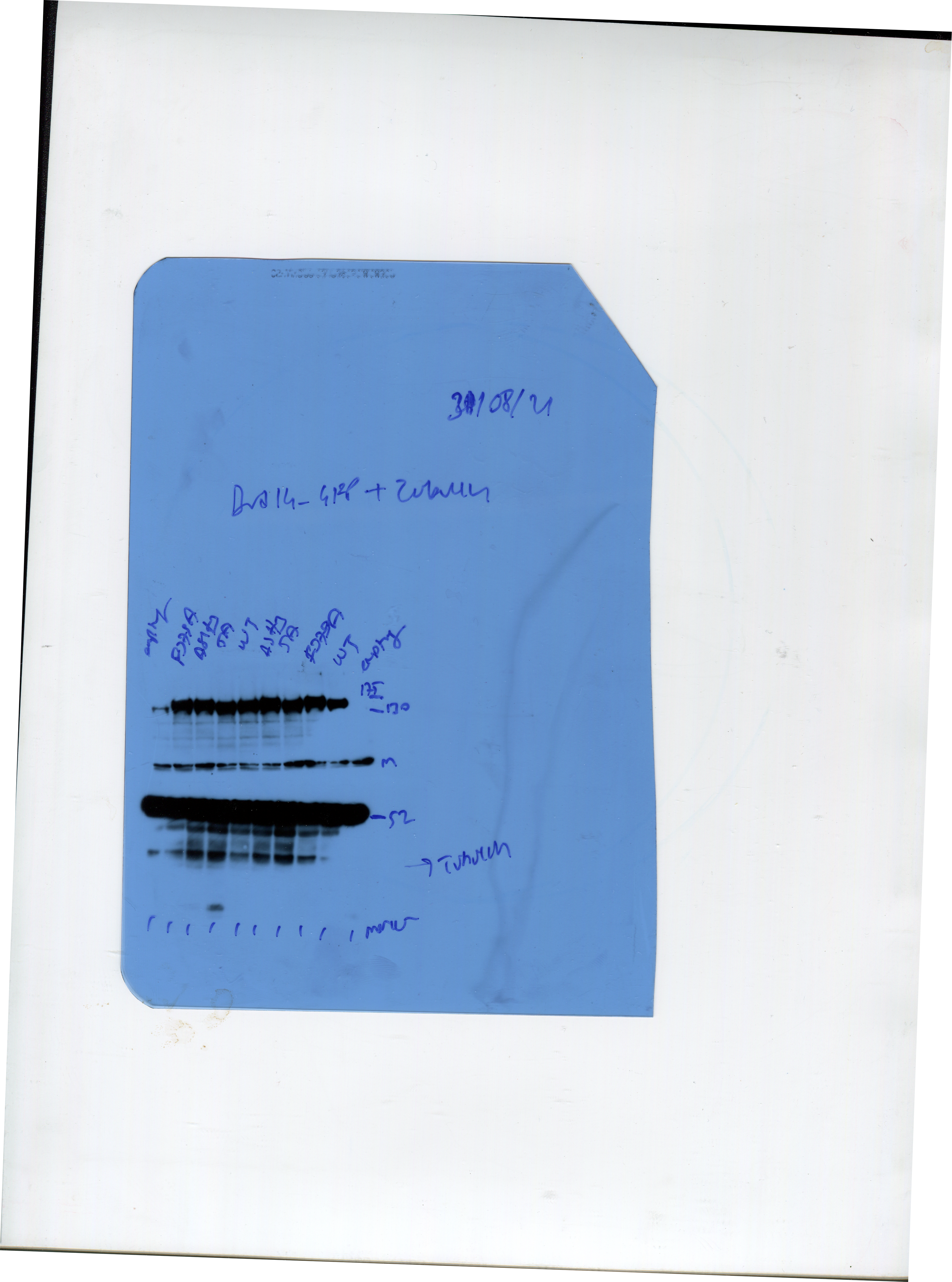

Supplement: Figure 3—figure supplement 1—source data 2. [file elife-72833-fig3-figsupp1-data2.zip › Figure 3 - Figure Supplement 1A-Source Data 2/Figure 3 - Figure Supplement 1A-Source Data 2A.tif]

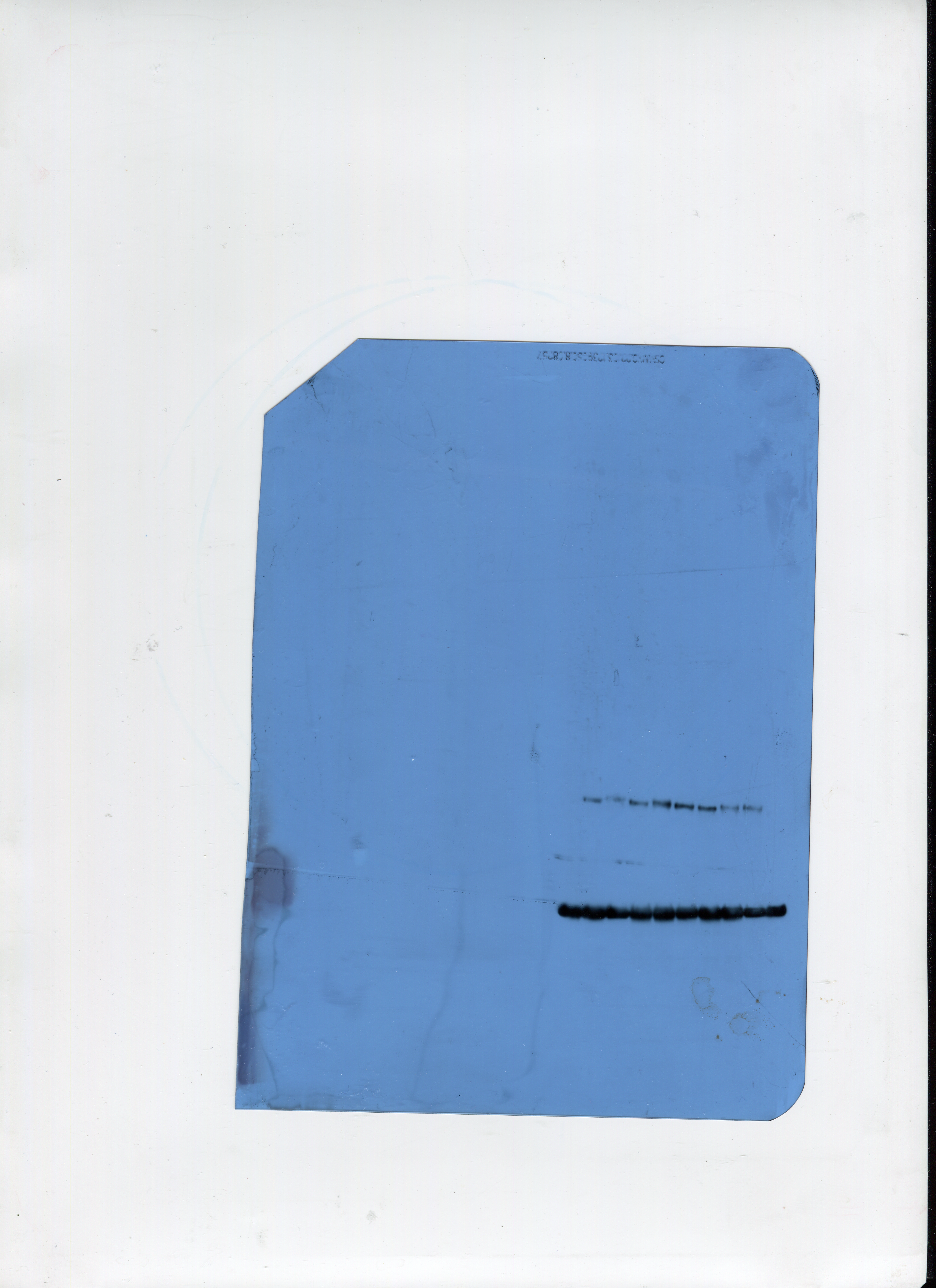

Supplement: Figure 3—figure supplement 1—source data 2. [file elife-72833-fig3-figsupp1-data2.zip › Figure 3 - Figure Supplement 1A-Source Data 2/Figure 3 - Figure Supplement 1A-Source Data 2B.tif]

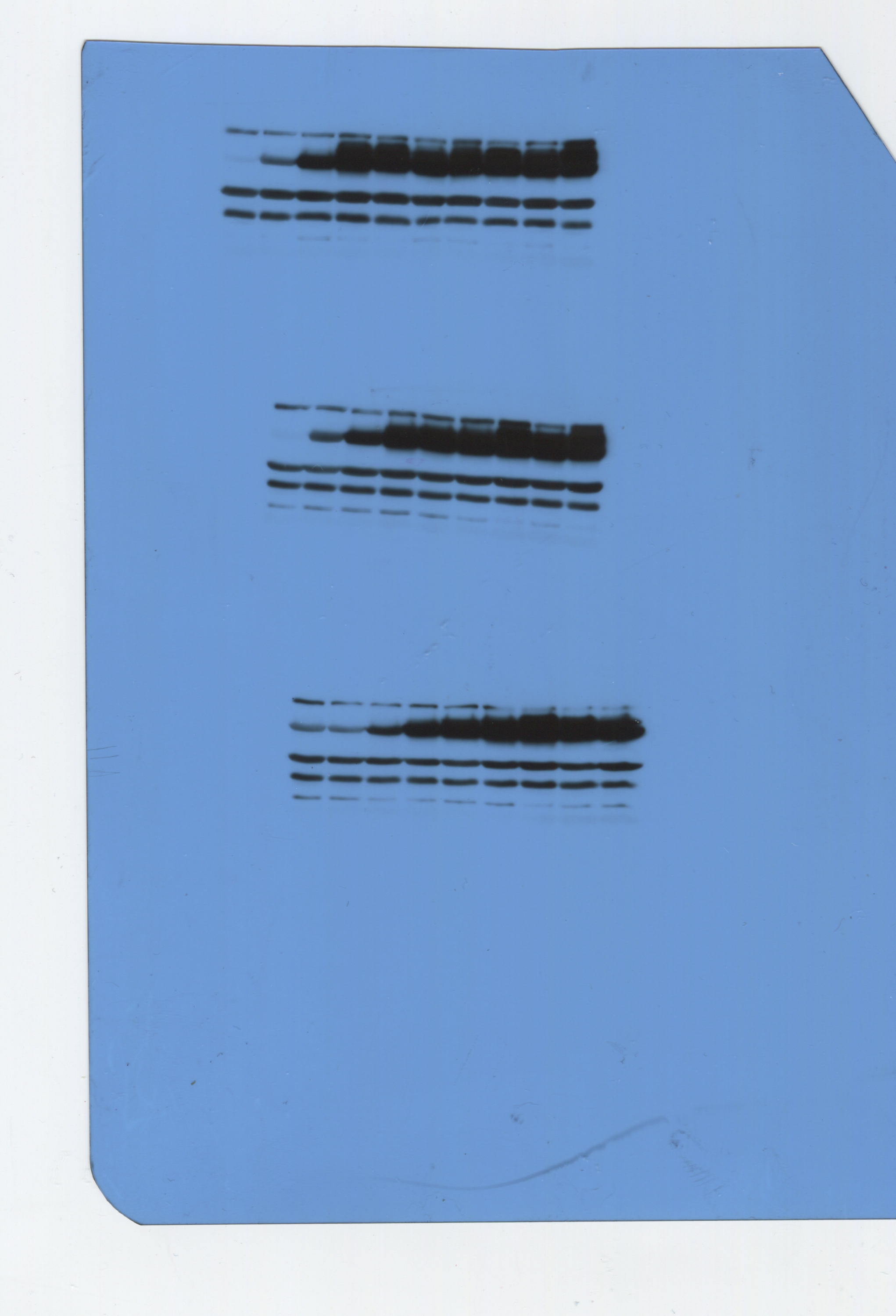

Supplement: Figure 7—source data 1. [file elife-72833-fig7-data1.zip › Figure 7A-7B-Source Data 2 /Figure 7A-7B-Source Data 2B.tif]

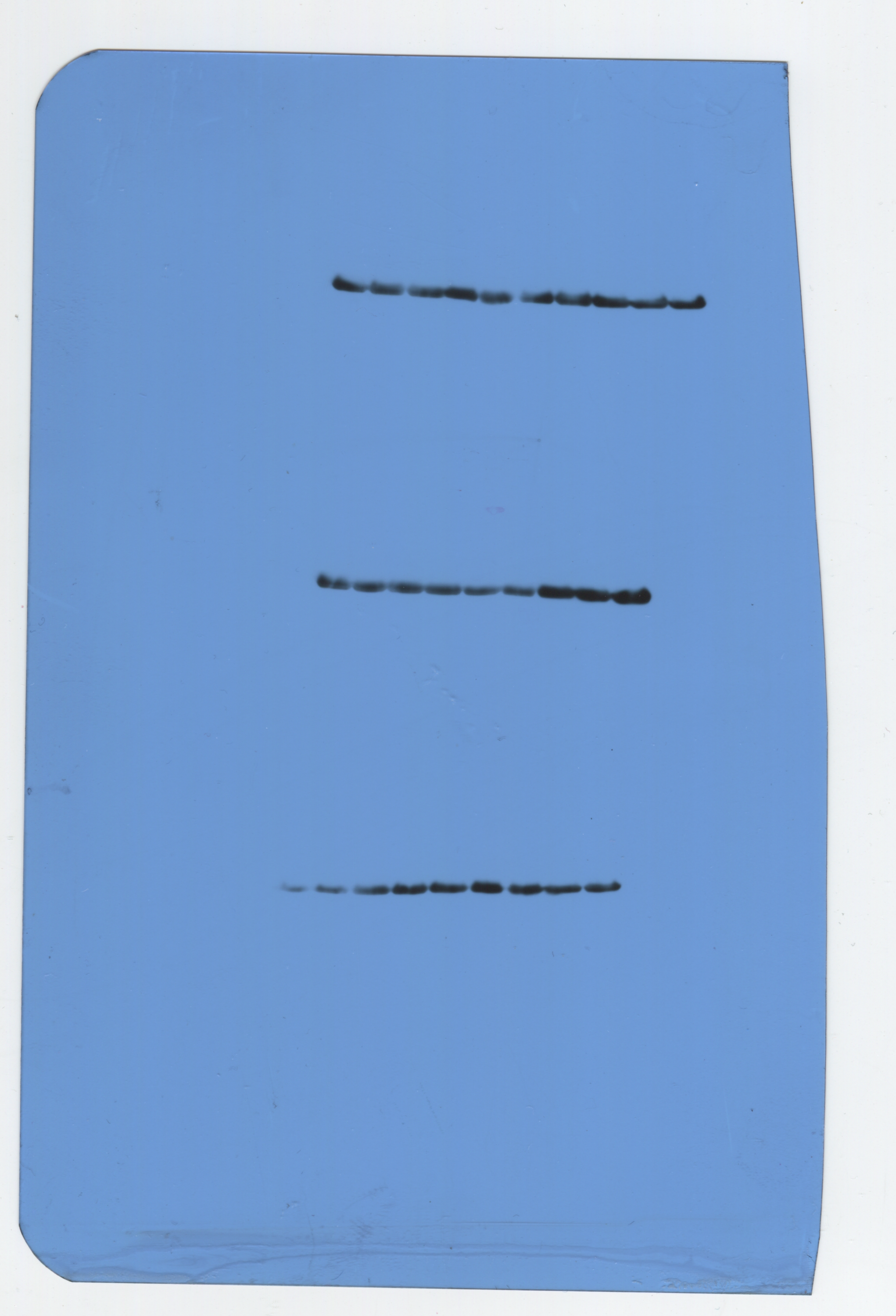

Supplement: Figure 7—source data 1. [file elife-72833-fig7-data1.zip › Figure 7A-7B-Source Data 2 /Figure 7A-7B-Source Data 2C.tif]

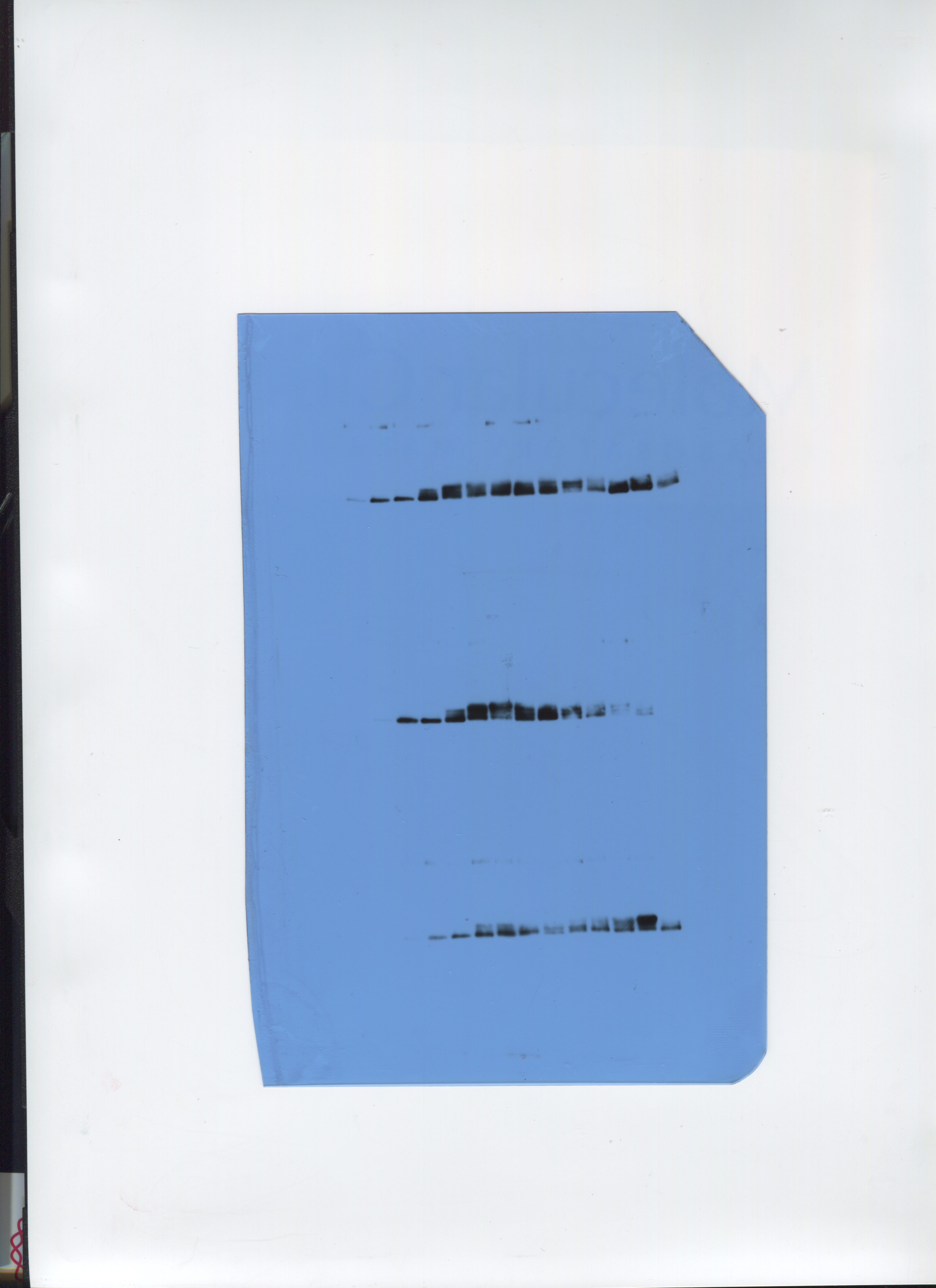

Supplement: Figure 7—source data 1. [file elife-72833-fig7-data1.zip › Figure 7A-7B-Source Data 2 /Figure 7A-7B-Source Data 2A.tif]

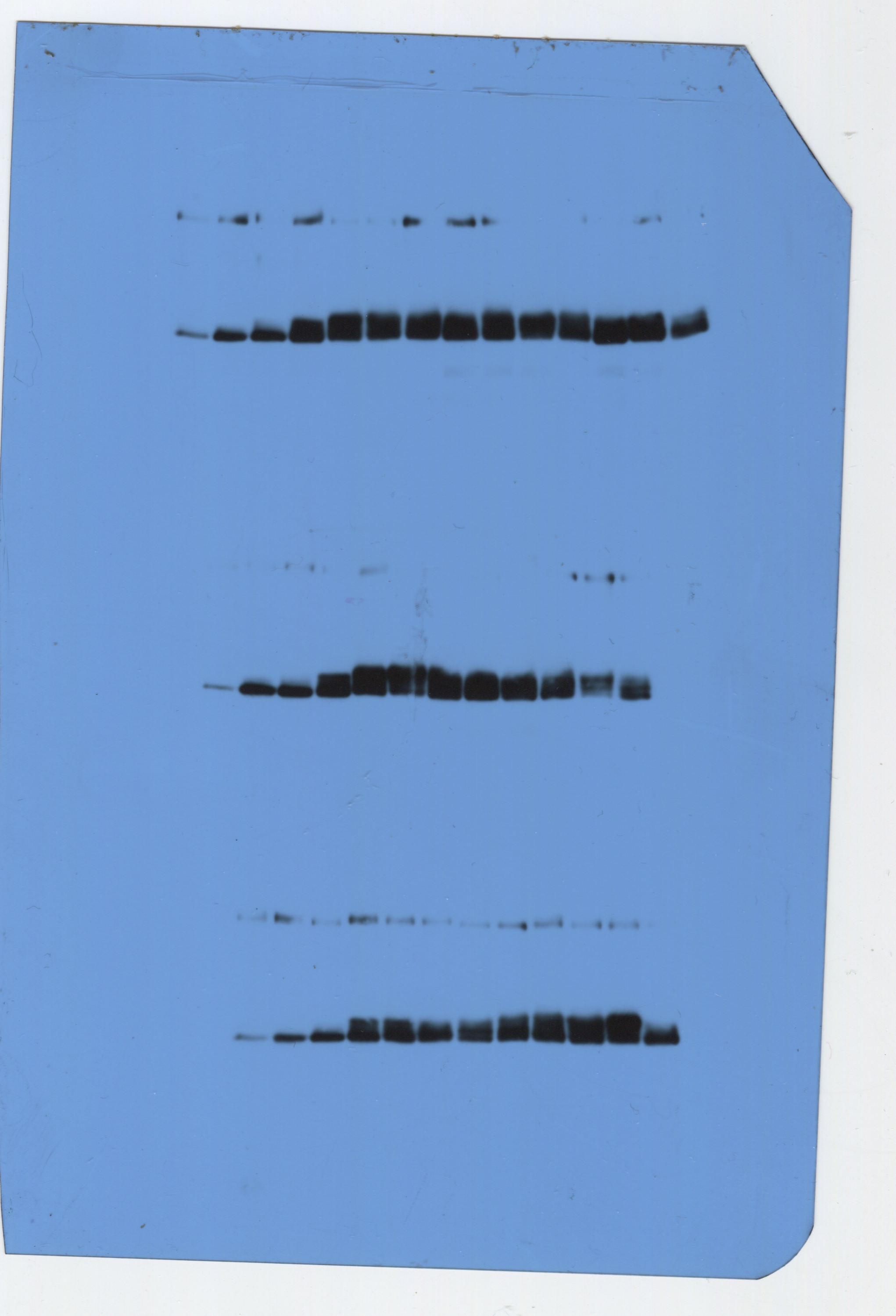

Supplement: Figure 7—source data 1. [file elife-72833-fig7-data1.zip › Figure 7A-7B-Source Data 2 /Figure 7A-7B-Source Data 2D.tif]

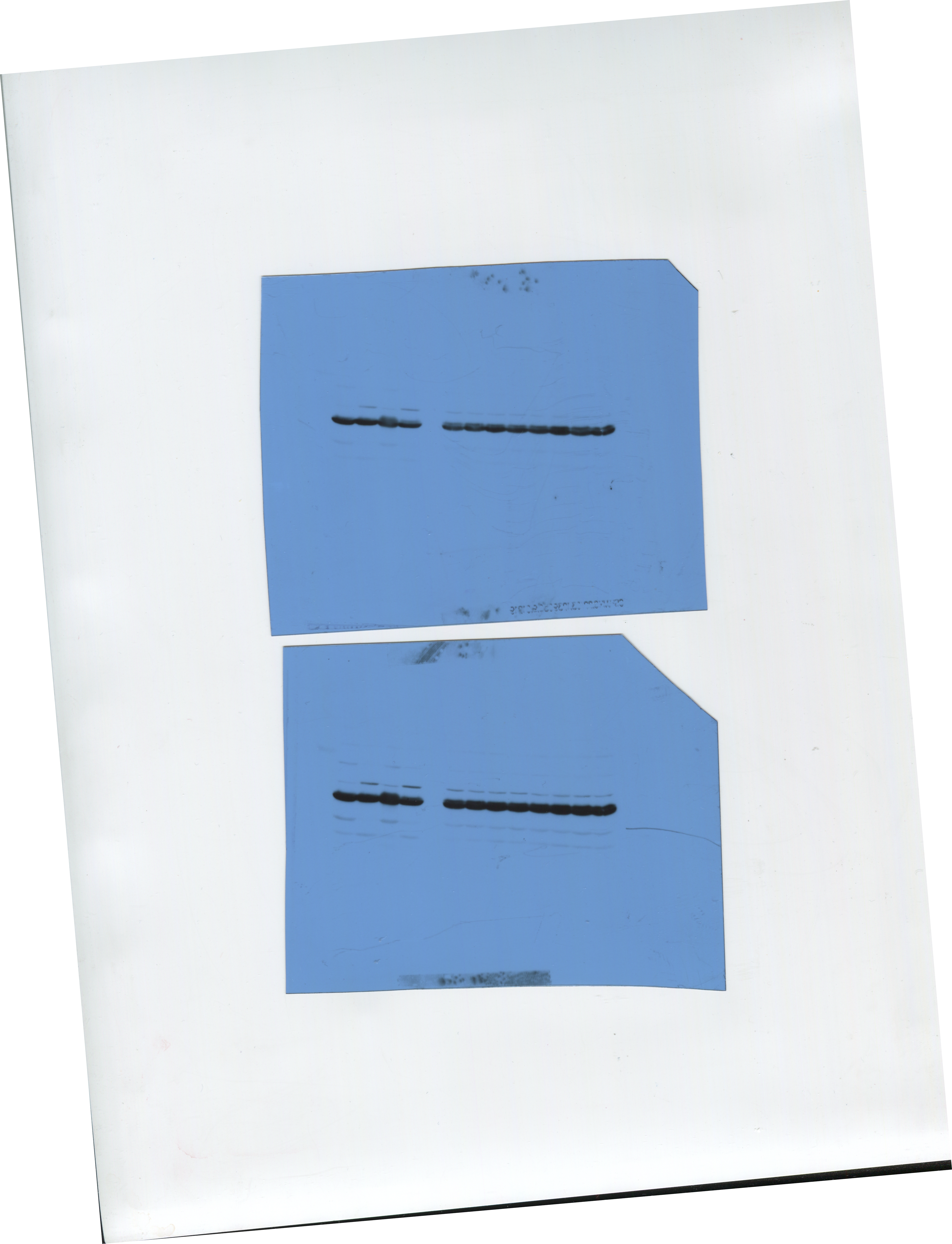

Supplement: Figure 7—source data 2. [file elife-72833-fig7-data2.zip › Figure 7C-7D-Source Data 2A-C/Figure 7C-7D-Source Data 2C.tif]

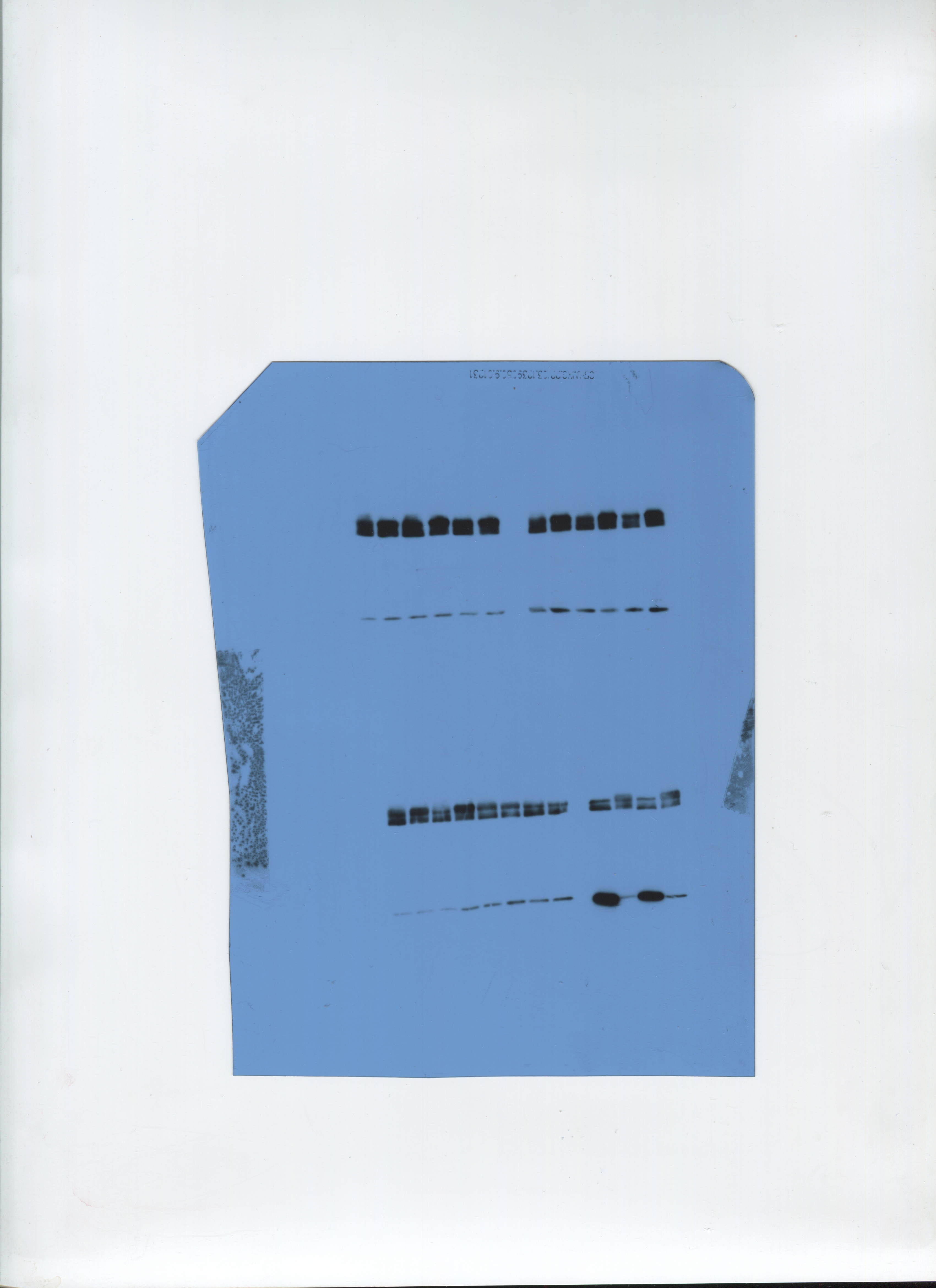

Supplement: Figure 7—source data 2. [file elife-72833-fig7-data2.zip › Figure 7C-7D-Source Data 2A-C/Figure 7C-7D-Source Data 2A.tif]

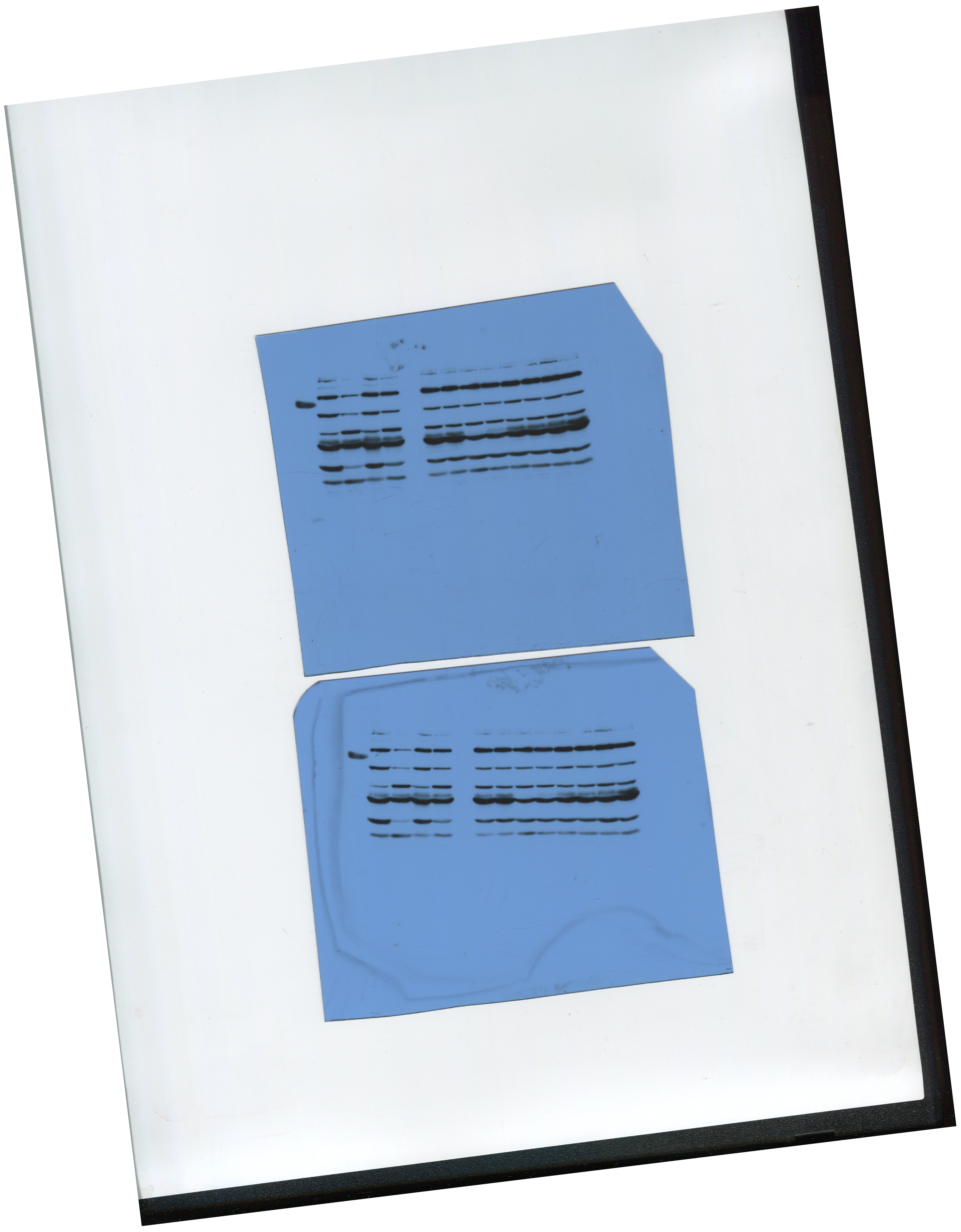

Supplement: Figure 7—source data 2. [file elife-72833-fig7-data2.zip › Figure 7C-7D-Source Data 2A-C/Figure 7C-7D-Source Data 2B.tif]

Figure 7 - Figure Supplement 1A-1B-Source Data1

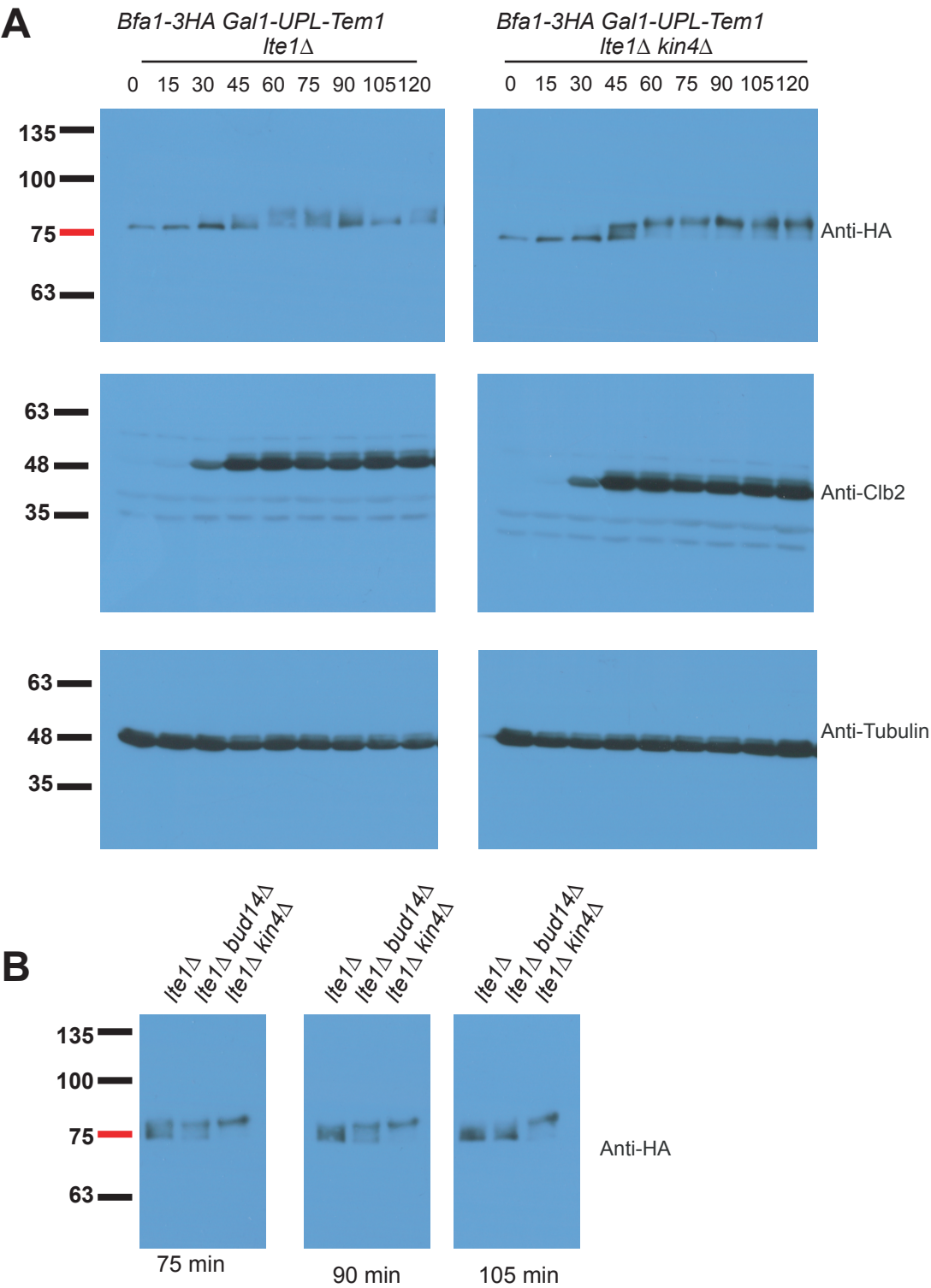

Supplement: Figure 7—figure supplement 1—source data 1. [file elife-72833-fig7-figsupp1-data1.pdf]

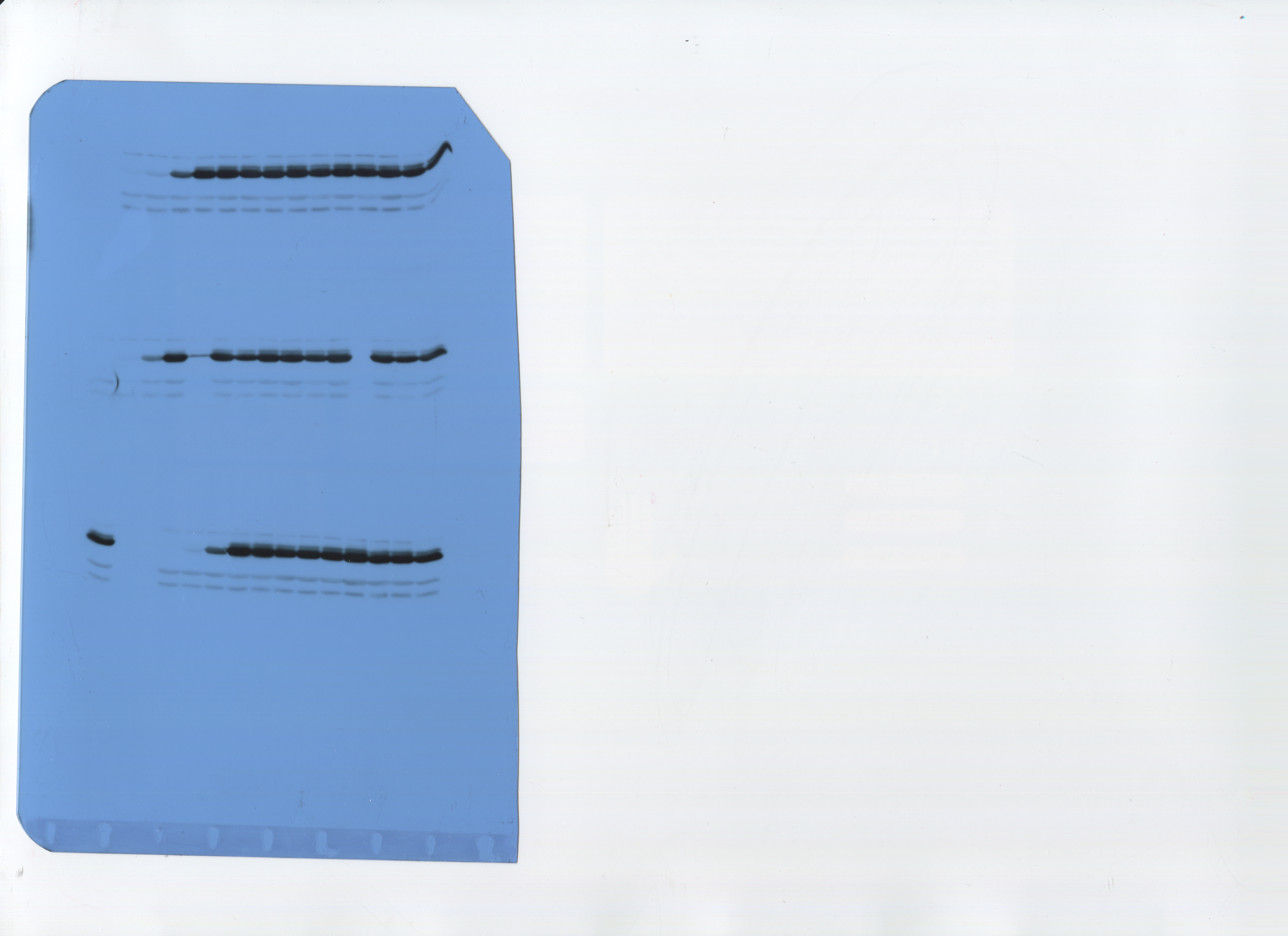

Supplement: Figure 7—figure supplement 1—source data 2. [file elife-72833-fig7-figsupp1-data2.zip › Figure 7- Figure Supplement 1A-1B- Source Data 2/Figure 7- Figure Supplement 1A-1B- Source Data 2B.tif]

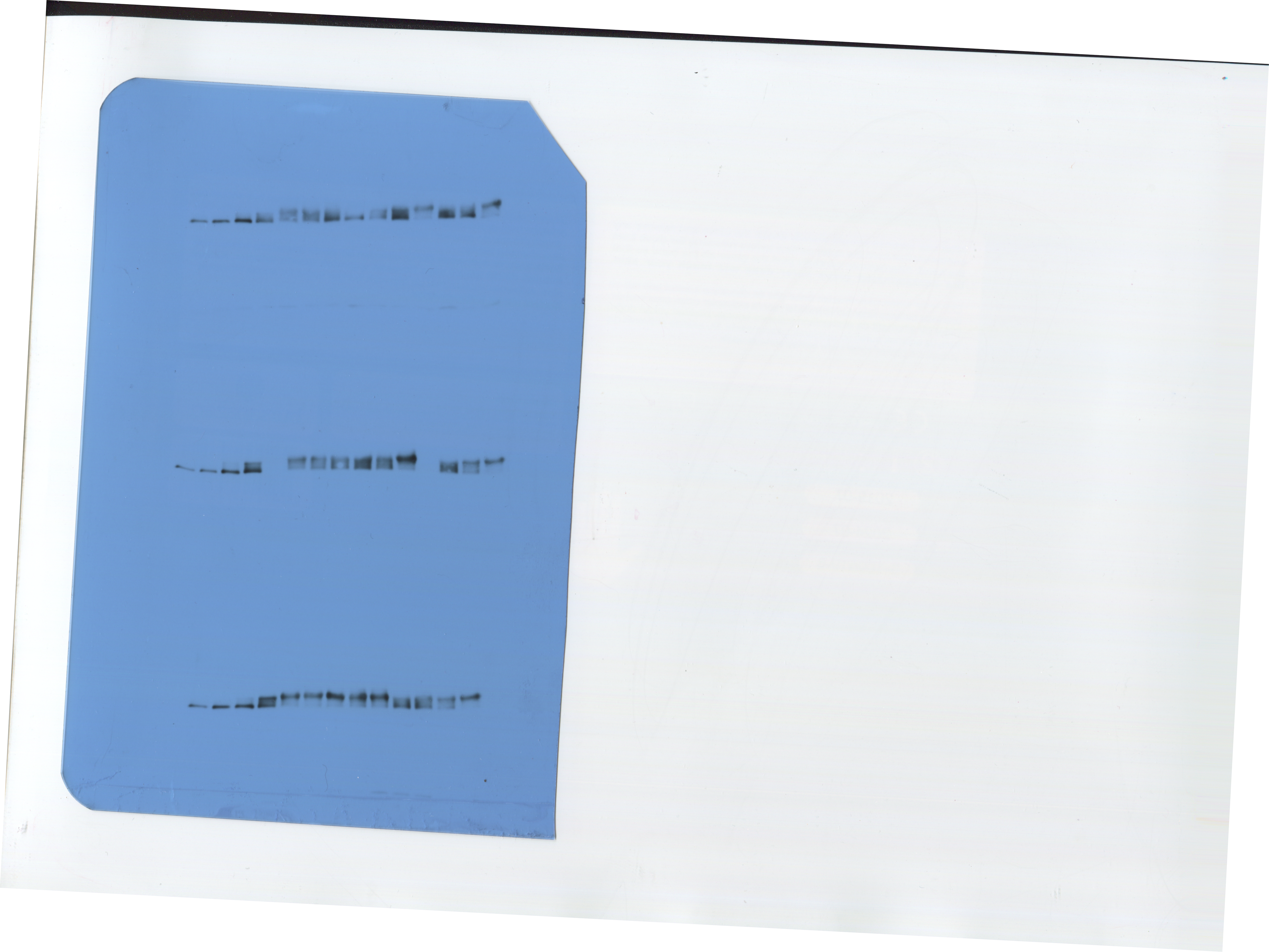

Supplement: Figure 7—figure supplement 1—source data 2. [file elife-72833-fig7-figsupp1-data2.zip › Figure 7- Figure Supplement 1A-1B- Source Data 2/Figure 7- Figure Supplement 1A-1B- Source Data 2A.tif]

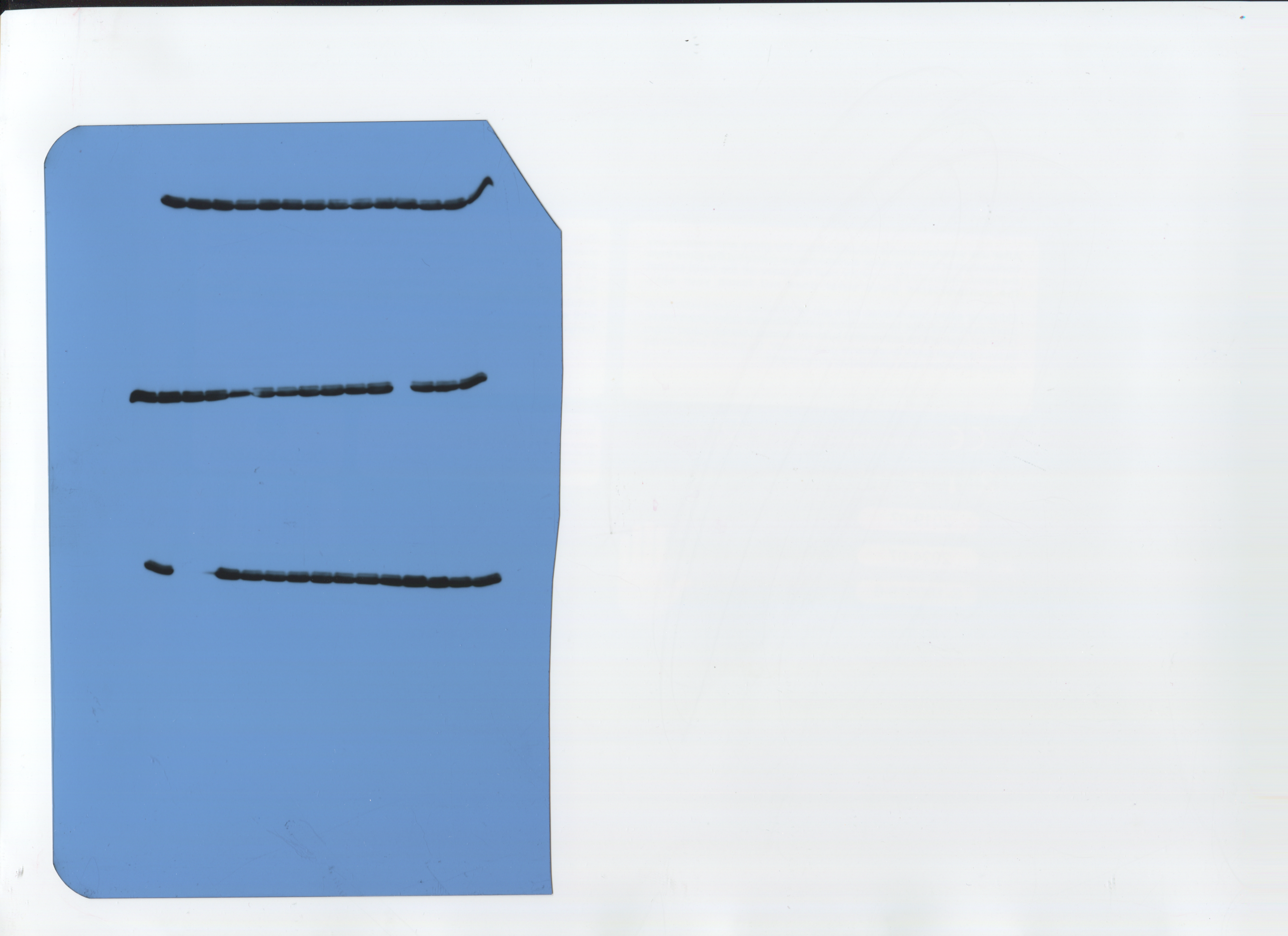

Supplement: Figure 7—figure supplement 1—source data 2. [file elife-72833-fig7-figsupp1-data2.zip › Figure 7- Figure Supplement 1A-1B- Source Data 2/Figure 7- Figure Supplement 1A-1B- Source Data 2C.tif]

Figure 7 - Figure Supplement 2B-Source Data 1

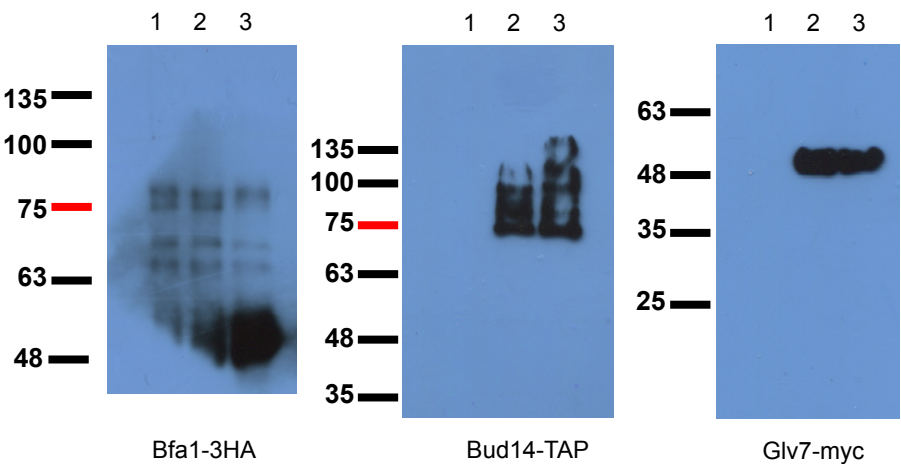

Supplement: Figure 7—figure supplement 2—source data 1. [file elife-72833-fig7-figsupp2-data1.pdf]

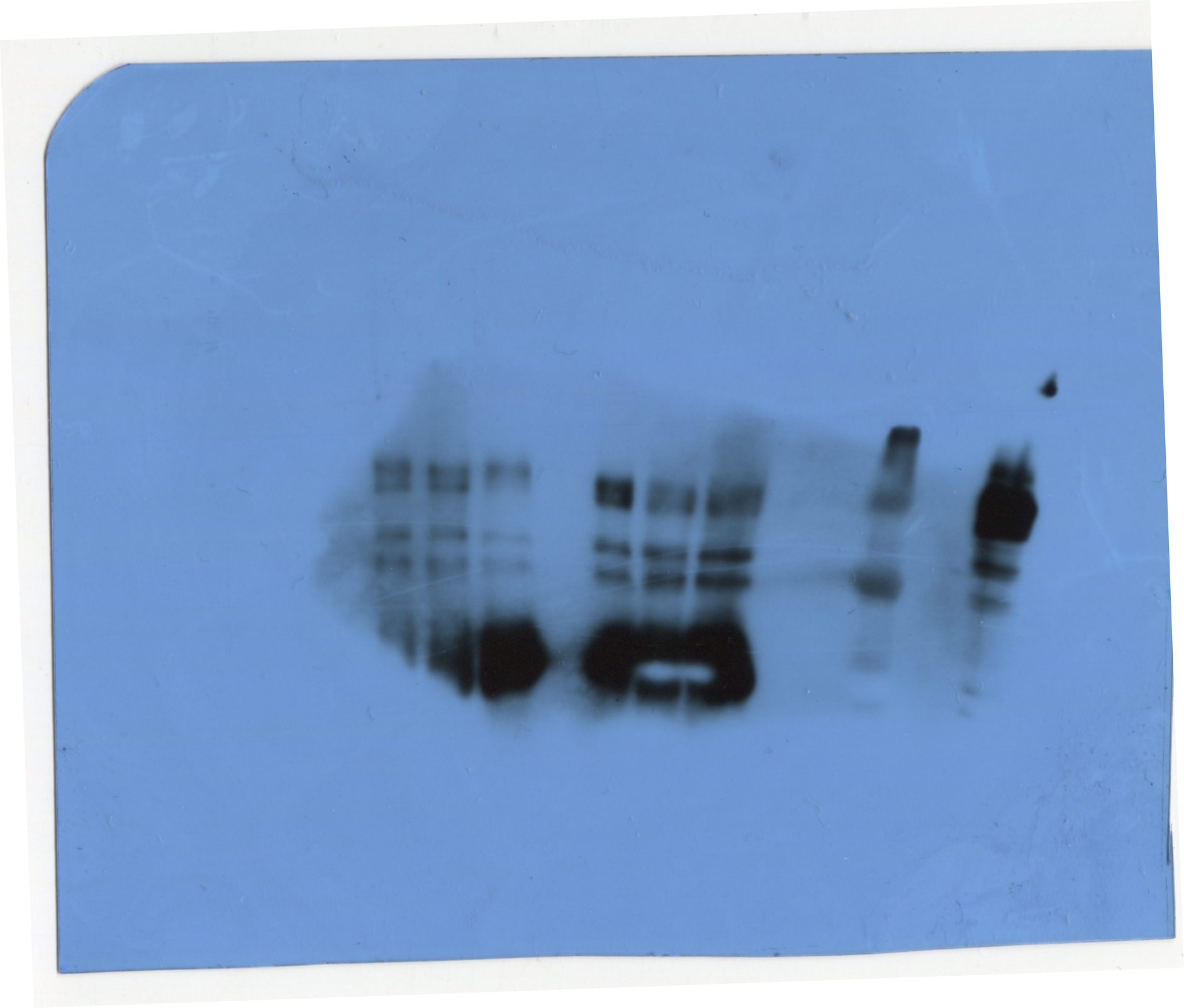

Supplement: Figure 7—figure supplement 2—source data 2. [file elife-72833-fig7-figsupp2-data2.zip › Figure 7 - Figure Supplement 2B-Source Data 2/Figure 7 - Figure Supplement 2B-Source Data 2A.tif]

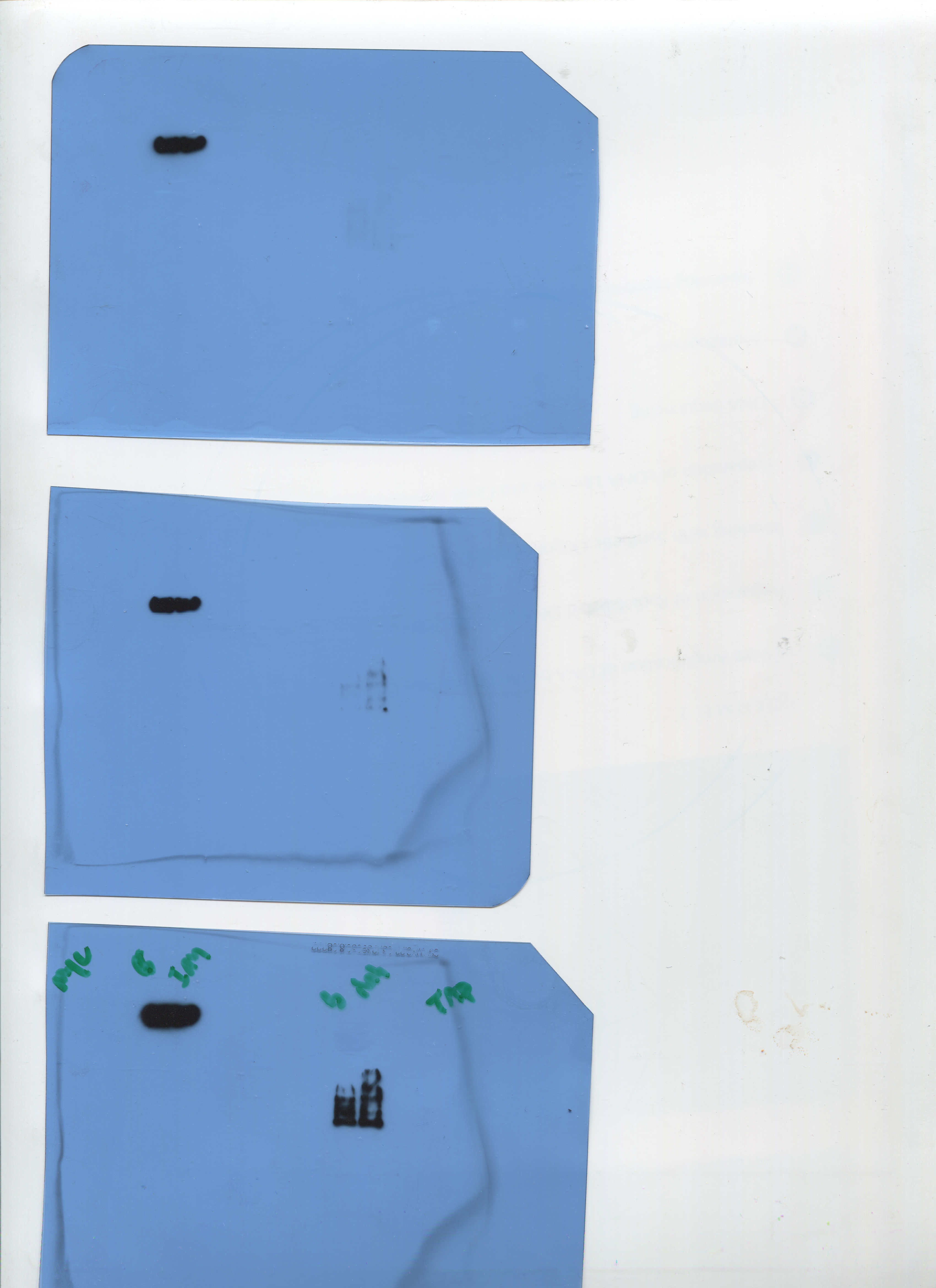

Supplement: Figure 7—figure supplement 2—source data 2. [file elife-72833-fig7-figsupp2-data2.zip › Figure 7 - Figure Supplement 2B-Source Data 2/Figure 7 - Figure Supplement 2B-Source Data 2B.tif]
